# A fair trade? Expert perceptions of equity, innovation, and public awareness in China’s future Emissions Trading Scheme

**DOI:** 10.1007/s10584-021-02961-0

**Published:** 2021-02-06

**Authors:** Jiangyue Joy Ying, Benjamin K. Sovacool

**Affiliations:** 1 Science Policy Research Unit (SPRU), School of Business, Management, and Economics, University of Sussex, Brighton, UK; 2 Center for Energy Technologies, Department of Business Development and Technology, |Aarhus University, Aarhus, Denmark; 3 Science Policy Research Unit (SPRU), University of Sussex, Jubilee Building, Room 367, Falmer, East Sussex BN1 9SL UK

**Keywords:** Climate policy, Carbon trading, Emissions trading schemes, Energy justice, Equity

## Abstract

How can the Chinese emissions trading scheme (ETS) be redesigned or improved to better address issues of fairness and equity, innovation and learning, and awareness and social acceptance? In order to meet its 2030 carbon emission reduction pledges, the Chinese government has announced plans for a fully implementable national carbon ETS after 2020. This scheme is set to become the world’s most significant carbon trading market and it could cover half of all Chinese CO_2_ emissions (as much as 4 billion tons of carbon dioxide). In this study, we qualitatively analyze the Chinese ETS through the lens of three interconnected themes—equity, innovation, and awareness—which are disaggregated into six specific dimensions. We then explore these themes and dimensions with a mixed methods and original research design involving a survey of 68 Chinese experts as well as 34 semi-structured research interviews with respondents from local governments, financial institutions, technology service companies, universities, industries, and civil society groups. We find that uneven economic and social growth could exacerbate any initial permits allocation scheme that could be a cornerstone for an ETS. Substantial technological and institutional uncertainties exist that could also hamper development and enforcement. Low or negative awareness among the public and private sector were identified as also being significant barriers for ETS implementation.

## Introduction

Emissions Trading Schemes (ETS) are expected to become one of the most cost-effective, or at least popular, mechanisms for mitigating carbon emissions in the future (WGEA 2016). Currently, at least 20 relatively mature ETS are operating across the European Union, Japan, New Zealand, United States, and Canada (Trotignon et al. 2011; Li et al. 2012; ICAP 2019). These operating carbon markets account for approximately 8% of global carbon emissions (ICAP 2019). Meanwhile, by 2020, the volume of global carbon trading is predicted to reach US$3.5 trillion (Wang et al. 2019), given both more countries participating in carbon markets as well as an increase in the volume of tradable credits.

As the world’s largest developing country, China is facing a monumental series of interconnected energy and climate challenges, including rising energy demand, deteriorating environmental quality, and eroding energy security (Dong et al. 2016; Wang et al. 2019; Yang et al. 2017; Zhou et al. 2019a, 2019b). Simultaneously, as the world’s largest carbon emitter, China’s response to climate action undoubtedly will shape global emissions trends (Liu et al. 2015).

Given these dynamics, China has agreed to a 2030 CO_2_ emissions peak target and CO_2_ intensity reduction pledge that is said to meet both the Copenhagen and Paris Agreements (Yang et al. 2017; Zhou et al. 2019a, 2019b). To fulfill these international pledges cost-effectively, China has stated a strong intention to establish a national carbon trading system (China’s ETS) over the course of its 13th Five-Year-Plan (FYP) spanning from 2016 to 2020 (NDRC 2016; Jotzo et al. 2018; Wang et al. 2019). This ETS is set to become the world’s most significant carbon trading market and is expected to cover 4 billion tons of CO_2_ (Dong et al. 2016; Springer et al. 2019; Goron and Cassisa 2017).

However, the ETS does not come without some challenges. As of 2020, the Chinese ETS remains in its infancy and it is expected that the first phase of trading will only involve the electric power sector (NDRC 2017). Moreover, over the past 15 years, the largest cap-and-trade scheme, the European Union’s ETS, has suffered challenges that cast doubt on its perceived effectiveness as a tool within China. Furthermore, whereas ETS schemes generally arise from liberalized energy markets in developed countries, China is known for its strong tradition of command-and-control policies within a communist marketplace (Duan et al. 2014; Zhang 2015; Zhang et al. 2017). As Rawski (1999: 148) explains, throughout much of its history, in China:

*Planners not only determine prices, quantities, and commodity flows, but also control the form of business organization, the appointment of managers, the selection of technologies, the calculation of wages, the formulation of business plans, and the scheduling of investment and production operations. At one time or another, China’s socialist planners have attempted to schedule meals for hundreds of millions of rural residents, to assign fuel quotas to each tractor and engine, and to manage the childbearing activity of individual women. Virtually every aspect of social life—even friendship, music, and language—became entangled with centrally imposed rules*.

Under this system, state-owned enterprises grew to account for more than 70% of investments in the country as well as more than 70% of credit. This created what theorists have called a “rigid central planning system” distinguished by the “preponderant role of state enterprises and considerable state interference in economic activity” (Borensztein and Ostry 1996).

Thus, given this history of state involvement, creating a beneficial and incentive-based CO_2_ market in China poses additional challenges in comparison to other countries (Jotzo et al. 2018). Moreover, although China’s NDC under the Paris Accord sets the goal of a peak in carbon emissions by 2030 and a proposed 60–65% reduction in carbon intensity, it may not be sufficient to catalyze economy-wide decarbonization. Duan et al. (2018) warn that “the probabilities of realizing the carbon emission-peaking target and non-fossil energy target are low” and conclude that additional policy efforts are necessary. Zhou et al. (2019a, 2019b) write that “numerous barriers exist that will need to be addressed through effective policies and programs in order to realize [China’s] potential energy use and emissions reductions.”

**Fig. 1:**
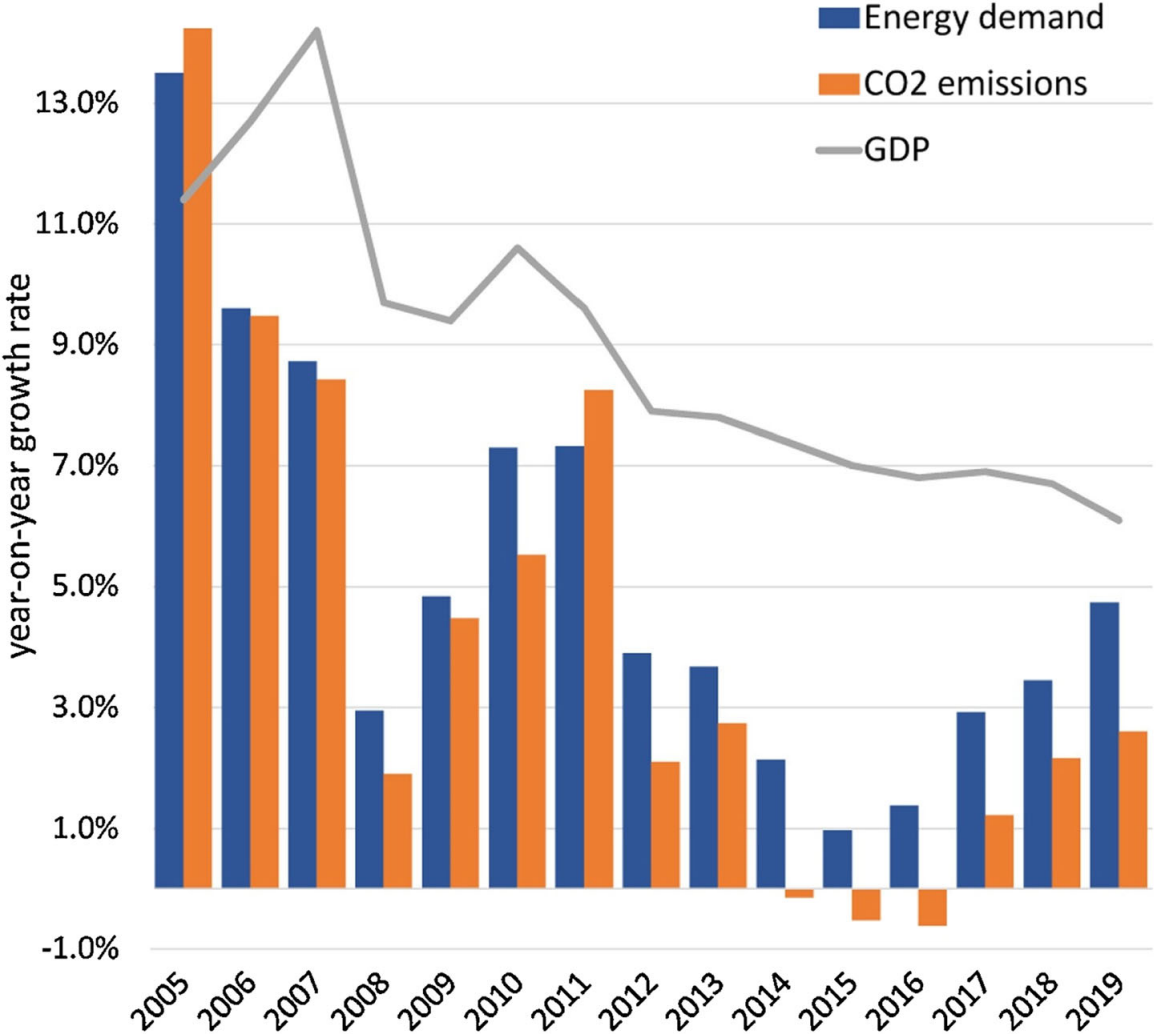
Annual growth in Chinese energy use, carbon dioxide emissions, and gross domestic product, 2005–2019 Source: Gosens and Jotzo (2020)

Perhaps, for these reasons, as Fig. 1 shows, China’s energy use and carbon dioxide emissions continue to climb. The global Covid-19 pandemic is making Chinese decarbonization even more difficult, as it has seriously disrupted domestic renewable energy manufacturing and exports (Sovacool et al. 2020) and it has reoriented investment into the fossil fuel industries, while support policies for renewable energy industries are absent from Beijing’s recovery program (Gosens and Jotzo 2020).

Therefore, it is imperative that China’s ETS appreciates the reality of Chinese emissions trends, and that it also anticipates potential challenges if it wishes to play a significant but effective role in global efforts to curb emissions trajectories (Jotzo et al. 2018). As Dong et al. (2018: 31) write succinctly, “it is crucial for China to allocate national carbon abatement targets fairly and efficiently.”

But how? In this study, we ask: how can the Chinese ETS be redesigned or improved to better address issues of fairness and equity, innovation and learning, and awareness and social acceptance? As an answer, we explore these themes and dimensions with a mixed methods and original research design involving a survey of 68 Chinese experts as well as 34 semi-structured research interviews with respondents from local governments, financial institutions, technology service companies, universities, industries, and civil society groups. The study begins by offering some brief background on the Chinese ETS as well as emerging problems related to environmental performance, industrial competitiveness, and governance. It then operationalizes the themes of equity, innovation, and awareness and explicates its research design, before moving to a discussion of results and findings. It concludes with implications for climate research and policy more generally.

## Background, thematic approach, and research design

This section offers some brief background to the Chinese ETS before explaining the thematic approach and research methods utilized in the study.

### A brief history of the Chinese Emissions Trading Scheme (ETS)

**Fig. 2:**
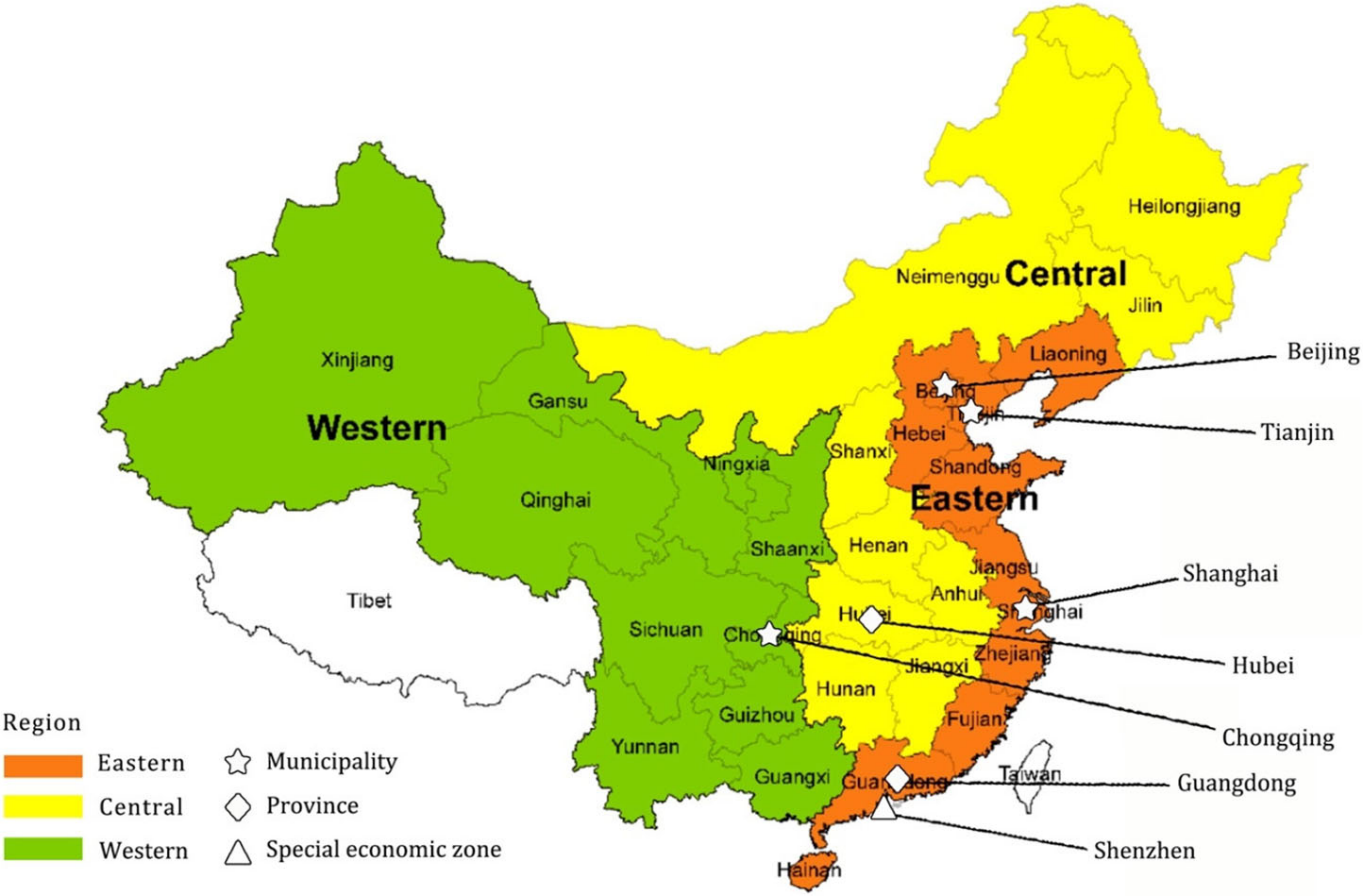
Map of China’s approved and launched seven ETS pilots. Source: Compiled by the authors based on Zhang et al. (2014) and Yang et al. (2016)

The history of emissions trading in China is concise but complex. In order to achieve reduction commitments cost-effectively, seven piloting ETS programs in China began implementation in 2013 at both the province and city level, including Beijing, Tianjin, Shanghai, Guangdong, Hubei, Shenzhen, Chongqing (NDRC 2016; Zhang et al. 2014; Chen et al. 2017). The seven ETS pilots are located in various parts of China with different industrial structures and development trajectories. The different colors in Fig. 2 refer to Eastern (developed), Central (sub-developed), and Western (less-developed) regions. The regions are fairly different in terms of geographical energy consumption models, resource endowment, historically cumulative emissions, and economic development (Chang et al. 2016; Fan 2018).

These seven pilot projects also have shown great differences in the design of core elements, such as allocation schemes, coverage thresholds, and emission control standards, reflecting their respective features and priorities in the localities shown in Table 1. Based on the comparison in Table 2, we can observe that most of the seven ETS pilots are located in relatively affluent regions in China, but the carbon emissions trading volumes of pilots in 2016 were apparently different. This disparity will complicate efforts to construct China’s ETS (Zhang et al. 2014).

**Table 1:** Features of China’s national ETS as announced in December 2017

Nationally determined contribution (NDC)	ETS timing and phases	Coverage and threshold	Allocation of emissions allowances
Stop the rise in absolute CO_2_ emissions around 2030. Reduce CO_2_ emissions intensity of the economy by 60–65% in 2030, relative to 2005. Increase the share of non-fossil fuels in primary energy consumption to around 20% in 2030.	Announced in December 2017: **First phase:** national emissions reporting framework (one year). **Second phase:** trial run (simulation)for the electricity sector (one year). **Third phase:** full operation in the electricity sector (around 2020) Gradual expansion to other industries (building materials; petrochemicals; chemicals; iron and steel; non-ferrous metal processing; pulp and paper; aviation).	**Electricity sector coverage:** around 3 GtCO_2_. **Full expected coverage:** around 5 GtCO_2_. **Threshold:** ETS covers companies with emissions above roughly 26,000tCO_2_ per year (annual energy consumption of more than 10,000t of coal equivalent).	**Freely allocated** to industry. **Output-based allocation** based on benchmarks (sub-sectoral performance standard) with ex-post adjustments.

Source: Jotzo et al. 2018

**Table 2:** Overview of China’s seven carbon trading pilots

	Beijing	Shanghai	Tianjin	Hubei	Guangdong	Shenzhen	Chongqing
**General information**							
Population (million) (2014)	19.6	23	13	57.2	104.3	10.4	28.9
GDP Per capita (2014, in CNY)	93, 213	90,092	99,607	42,613	58,540	136,947	42,795
Total CO_2_ emissions (in Mt)	188.1	297.7	215	463.1	610.5	153	243.1
Starting date	Nov. 2013	Nov. 2013	Dec. 2013	Apr. 2014	Dec. 2013	Jun. 2013	Jun. 2014
Carbon intensity reduction target (by 2020)	20.50%	20.50%	20.50%	19.50%	20.50%	21%	19.50%
**ETS size**							
Covered CO_2_ emission (MtCO2e)	50 (2017)	158 (2018)	165 (2017)	257 (2017)	422 (2018)	31.45 (2015)	100 (2018)
Scope (2018)	Industry; Power; Buildings; Transport	Industry; Power; Buildings; Transport; Aviation	Industry; Power; Buildings	Industry; Power	Industry; Power; Domestic Aviation	Industry; Power; Buildings	Industry; Power
Number of covered entities (2018)	943	298	109 (2017)	344 (2017)	288	794 (2017)	195
Average carbon price (2018)	37.97 CNY (USD 5.74)	37.99 CNY (USD 5.74)	11.58 CNY (USD 1.75)	21.23 CNY (USD 3.21)	15.10 CNY (USD 2.28)	24.47CNY (USD 3.70)	4.36 CNY (USD 0.66)
Allocation methodology	Free allocation	Free allocation Auctioning	Free allocation	Free allocation	Free allocation Auctioning	Free allocation	Free allocation
Emissions trading volumes (10,000 tons) (2016)	242.92	415.57	31.05	1111.81	1396.78	1102.07	46.02

Source: Compiled by the authors based on Tanpaifang (2016), ICAP (2019), and Zhang et al. (2014)

Although China’s pilot systems have developed rapidly, they are still far from complete or even sufficient. The pilots were supposedly intended to identify and resolve the institutional and technical challenges of the application of a market-based instrument in a strongly centralized political environment (Chen et al. 2017; Lo 2013). Furthermore, the pilots have been prone to multiple problems and barriers, which fall into three classes of environmental, economic, and programmatic aspects.

In the *environmental* domain, one class of barriers remain in the realm of pollution and low-carbon energy. Lin and Jia (2020) found that the ETS was, counterintuitively, having a prophylactic effect on renewable energy. They concluded that unless renewable energy systems receive additional subsidies, the ETS will continue to increase costs and decrease generation. Gao et al. (2020) modeled the effects of the ETS on 28 industries in 30 provinces during 2005–2015. They found that the ETS had a greater effect on the mitigation of production-based emissions than consumption-based emissions, and, troublingly, that it encouraged the outsourcing of emissions from pilot areas to non-pilot areas, resulting in carbon leakage and the “pollution haven” effect. This is troubling as it implies the Chinese ETS is not eliminating emissions, just moving them outside of the trading region. Chen et al. (2020) write that the ETS could further promote regional unevenness rather than promote leapfrogging, a concern that as carbon prices increase, Jiangsu and Shanghai will in particular suffer potential losses.

In the *economic* domain, another realm of barriers relate to industrial performance, employment, competitiveness, and poverty. Modeling from Zhang and Duan (2020) suggests that China’s pilot ETSs have exerted a negative impact on gross industrial output value (GIOV) and employment, which both declined in pilot areas. Similarly, Zhang et al. (2019) express concerns that the ETS is depressing industrial competitiveness and manufacturing, decreasing the production outputs of key industrial subsectors. Zhang and Zhang (2020) note that the ETS could even affect significantly patterns of rural development and poverty, impacting household income inequality.

In the *programmatic* domain, a final set of barriers relate to programmatic design and institutional governance. Hu et al. (2020) suggest that while the ETS pilot policy has seen energy consumption in some regulated industries decrease by 22.8% and CO_2_ emissions by 15.5% compared to those in nonpilot areas, it has performed far better in areas with high levels of enforcement and marketization. Its effectiveness, in other words, is conditional on governance. Yet in this domain, Dong et al. (2019) argue that the ETS has been prone to constantly changing quotas and high levels of uncertainty, given a lack of detailed guidance in national policy; lack of transparency in information about emissions trading volumes and leakage; and weak regulation and legal support. Lo and Chen et al. (2020) add that the pilot ETS has only reinforced the domination of state actors and not fully engaged with compliance enterprises (i.e., polluters) or financial institutions. Ji et al. (2020) critique that the ETS has resulted in carbon prices that fluctuate greatly, and are set to be too low, given that an oversupply of allowances is leading to low auction prices. Even a mix of survey and modeling evidence from Gallagher et al. (2019) has indicated that the ETS in China needs reform, noting that “the effectiveness of the ETS also depends on the full implementation of power sector reform, as do other fiscal policies like the feed-in tariffs" and that “the new national ETS for the power sector, while important, is not currently sufficient to induce major emission reductions because of the anticipated low prices and narrow sectoral coverage in the near term.”

With such issues in mind, we sought to develop a thematic approach in our study revolving around the three important considerations of equity, innovation, and public awareness.

### Thematic approach: equity, innovation, and awareness

The first leg of our tripartite thematic approach centers on *equity*. Equity principles are often used in international climate negotiations, with the burden sharing of emissions reductions seen as related to both the current and future economic development potential of a country or a region (Posner and Sunstein 2007; Reen 2009; Mumma and Hodas 2008; Yang 2011; He et al. 2006). As a result, scholars have expounded and then embedded principles of equity across perspectives as varied as the per capita principle (Kverndokk 1995), the nationalism principle (Young and Wolf 1992; Blanchard et al. 2001), and the polluter pays principle (OECD 1974). Rose (1992) and Rose et al. (1998) have even categorized equity principles into three separate categories: allocation-based, outcome-based, and process-based. Ringius et al. (2002) further detailed three levels of equity principles: justice and fairness concepts, the burden sharing formulae or rules, and the criteria and indicators.

The second leg of our thematic approach centers on *innovation*, including learning and technical development. In the context of an ETS, one of the appealing features is that this market-based mitigation instrument might be able to stimulate companies to apply innovative or resource-saving technologies whereby the flexibility and incentives are brought about by carbon pricing (Lutz et al. 2005; Shi et al. 2018; Wu et al. 2005). Subramanian et al. (2007) even explored the influence of the amount of carbon quotas on different types of companies’ innovation inputs and outputs. The results showed that changing the number of carbon quotas has a far greater impact on the investment flowing into innovation. This innovation, in turn, could be an effective means to combat climate change and achieve reduction in carbon emissions (Ding et al. 2016; Zhou et al. 2010).

The third and final leg of our thematic approach is that of public *awareness* and social acceptance. Studies have also confirmed that public awareness toward climate change is a significant factor in reducing carbon emissions (Chang et al. 2012; Environomist 2017). Meanwhile, carbon emissions from household consumption have become a significant driver of emissions, especially in developed countries (Hårsman and Quigley 2010). Although technological innovation can remediate some of these problems, it cannot fully eliminate the impacts of unsustainable lifestyle (Chen 2014). As the carbon emissions of individuals are difficult to track, record and quantify, the public thus has to be made to understand the respective influence of their behavior on climate change, and change the ways that they live in order to reduce carbon emissions (Fisher et al. 2000). Generally speaking, people who are aware of the factors and impacts of global warming are more inclined to support climate change mitigation policies (Fisher et al. 2000). In addition, as carbon pricing schemes generally cause increasing prices and financial burdens for households, they can result in direct citizen opposition (Rudolph 2015).

Synthesizing from these broad bodies of work, Table 3 presents our overall thematic approach, resting on the three themes of equity, innovation, and awareness, which are then disaggregated into six specific dimensions. We then proceeded to test these different components with a mixed methods and original research design.

**Table 3:** The key considerations of an ETS

Theme	Dimension	Explanation
*Equity*	Equitable allocations	The distribution of initial permits allocation is the first phase of the design and implementation of an ETS. However, the initial allocation of emissions permits is controversial, because it determines the regional distribution of this cost burden. Equity considerations mediate any attempt to distribute the responsibility of emissions reduction or the entitlement of emitting.
	Equitable procedures	
*Innovation*	Innovative technology	Technological innovation is often perceived as an effective means to combat climate change. Many countries have begun devising and employing innovation-driven development strategies. ETS, as an artificially created market, also require careful institutional designs that will continuously evolve.
	Innovative institutions	
*Awareness*	Public awareness	Public awareness and participation are a significant driver of environmental policies. Meanwhile, public awareness and acceptance of a climate policy can create political support for emissions reductions. An ETS also needs to adhere to the principle of public participation in environmental public governance.
	Public participation	

Source: Authors

### Research design: a survey and semi-structured research interviews

With our thematic approach in place, the authors distributed an expert survey via Tencent’s survey platform. The survey consisted of 11 separate questions divided into four distinct sections. First, participants were asked to rank their reasoning regarding the necessity and challenges in implementing China’s ETS. The second section explored the ways in which China’s ETS related to equity considerations. The third part related to innovative institutions and innovative technologies. The fourth part related to public awareness and future expectations. The survey was distributed online and completed by 68 expert respondents. A copy of the semi-structured survey is offered in Appendix A.

The authors then conducted qualitative interviews with 34 respondents who were either experts or scholars engaged in carbon emission trading schemes in China. These interviews were conducted by a native Chinese speaker, but then translated into English. All interviews were then fully transcribed, and then fully coded, including frequency and content analysis of themes arising from within the interview statements. Table 4 shows the specific institutional affiliation of all respondents along with their unique respondent number. As the data shows, respondents came from a diverse but reflective mix of institution types, including local governments, financial institutions, technology service companies, universities, industries, and civil society groups. A copy of the interview questions in both English and Chinese, which followed a similar structure to the survey, is offered in Appendix B.

**Table 4:** Respondent numbers and institutional affiliation of expert interviews

No.	Institution type	Institutions
R1	Governments	Sichuan Provincial Development and Reform Commission, Department of Resource and Environment
R2		The People’s Government of The Tibet Autonomous Region, China
R3		The people’s Government of Sichuan province (地方金融监督管理局)
R4		Sichuan Provincial Development and Reform Commission
R5		Chengdu Municipal Development and Reform Commission
R6		Sichuan Academy of Environmental Policy and Planning
R7		The National Carbon Market Capacity Building Center (Chengdu)
R8	Monitoring and verification	China Quality Certification Centre (CQC), Chengdu Branch
R9		Shanghai Treasure Carbon New Energy Environmental Protection Technology Ltd.
R10		Clean Development Mechanism in Sichuan, China
R11	Exchanges and financial institutions	Sichuan United Environment Exchange
R12		China Hubei Emission Exchange
R13		Singularity Financial Limited (Hongkong)
R14	Information technology and services company	Sino-Carbon innovation and investment Co., Ltd (Beijing) Interviewee 1
R15		Sino-Carbon innovation and investment Co., Ltd (Beijing) Interviewee 2
R16		Xiong-An Green Development Research Institute (XA-GDRI)
R17		Chongqing Real Carbon Trading
R18		Sichuan PTC Technology Co., Ltd
R19		Sichuan Atract Cloud Energy Technology Services Co., Ltd
R20	Academia	Southwestern University of Finance and Economics, Institute of Chinese Financial Studies
R21		Sichuan Academy of Social Sciences, Macro Development Institute
R22		University of Lisbon, Climate Change and Sustainable Development policies, Institute of Social Sciences
R23		The Chinese University of Hongkong, School of Architecture
R24		Sun Yat-sen University, School of Geography and Planning
R25		Beijing Forestry University, International Institute of Applied System Analysis (IIASA)
R26		Beijing Institute of Technology, Center for Energy and Environmental Policy Research
R27	Non-governmental organizations	World Resources Institute (WRI) – Beijing, China
R28		SHAN SHUI Green Carbon Technologies Co., Ltd
R29		Green Earth Protection Technology Co., Ltd
R30		Inner Mongolia Environmental Sciences
R31		Idea Carbon
R32		Carbon Vision
R33	Emission entities	State Grid Sichuan Electric Power Company
R34		Hebei Jinyu Dingxin Cement Co., Ltd

Source: Authors

Although we consider that our study has a greater degree of triangulation given it relies on mixed methods (Sovacool et al. 2018)—a textual survey instrument with respondents completing the process privately and a verbal instrument of semi-structured interviews with respondents completing the task publicly with the interviewee—it nevertheless has some limitations. Our sample of respondents was a purposive one, rather than a fully representative one. Although all of our respondents classified themselves as experts when they participated, they will nevertheless have varying degrees of knowledge about the ETS, access to information, and perceptions about how it can be improved. Their responses will therefore vary with their particular background and experience—something that we maintain better reflects the messy reality of energy and climate policymaking. Moreover, many respondents that we approached (such as those on the NDRC) declined our invitation, and thus we were limited by the fact that only some of the respondents participated. Lastly, we did not make an attempt to weight, correct, normalize, or problematize interview and survey responses across our methods, to avoid censoring our results and discussion. In sum, our study helps “open up” discussions about the Chinese ETS by revealing stated preferences in all of their complexity, without prompting, and without overly framing questions or nudging respondents to answer in a certain way, rather than “close it down.”

## Equity, innovation, and awareness in the future of Chinese emissions trading

**Fig. 3:**
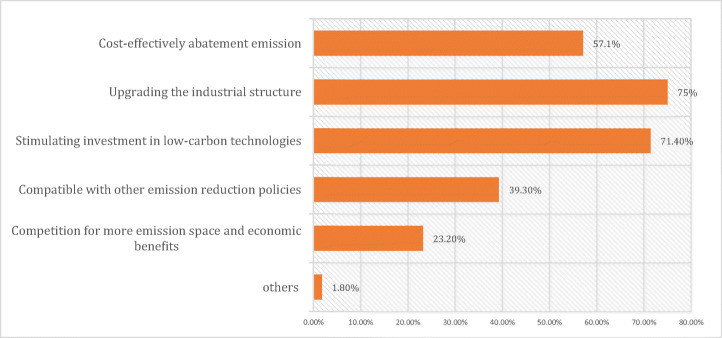
Survey responses about the purpose of implementing China’s ETS (n=68) Source: Authors

Most participants—slightly more than 90%—agreed upon the necessity of China creating a national carbon emission trading system. If China succeeds in utilizing the ETS effectively, the literature suggests not only economic benefits will be accrued but energy efficiency will also be bolstered (Wang 2013; Weng and Xu 2018). Participants were asked in the survey, *“What is the main purpose for China to implement the national ETS*?” Figure 3 represents that the most prominent answer, stated by 75% of respondents, was its ability to eliminate overcapacity and to upgrade Chinese industrial structure. Moreover, 71.4% of respondents to the survey also agreed that encouraging enterprises to invest in new abatement technologies would be greatly advantageous for the implementation of an ETS. Nonetheless, challenges concerning equity, innovation, and awareness strongly emerged from our findings as well. We discuss each in the remainder of this section.

### The equity dimensions of China’s ETS

**Fig. 4:**
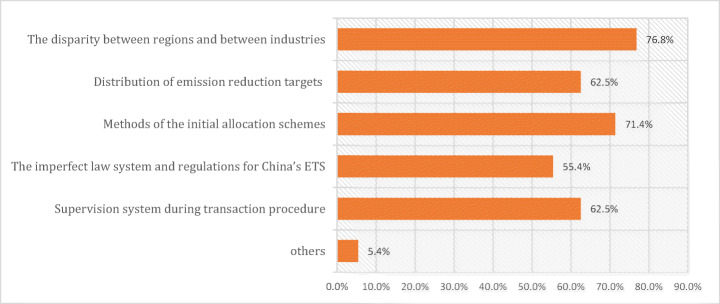
Survey responses reflecting on the equity and justice challenges within the Chinese ETS (n=68) Source: Authors

Issues of equity were confirmed as a pronounced and persistent challenge by multiple respondents. Regional equity will need to be considered carefully in future system design, because current permit allocation schemes could interact adversely with existing inequalities (Zhang et al. 2016). As R5 put it, *“The development of China’s ETS will first require addressing associated equity issues. Without an equitable and fair market, neither can the reduction targets be achieved, nor the unified ETS can operational.*” Overall, Figure 4 shows five different aspects of equity—disparity, distribution of targets, allocation, regulation, and system suspensions and lack of supervision—that were mentioned by at least *half* of all survey respondents.

Indeed, two particular equity challenges seem especially salient: the allocation of credits, and the equity of trading procedures. First, the allocation of permits was mentioned as a key determining factor in whether the ETS would be equitable as well as whether it would succeed or fail. In other ETS, such as those in Europe and California, policies have involved putting an absolute cap on emissions, which is reduced over time. China, however, uses a different approach, relying on a rate-based limit for its ETS. This establishes a limit put on the amount of CO_2_ allowed per unit of output. Each power company would be allocated a certain number of credits, depending on how much electricity it produces. If it emitted less than this set quota, it could then sell that surplus to another firm. However, China has generally resisted setting absolute emission caps in its climate pledges, instead opting for intensity-based targets to cut emissions per unit of GDP (Timperley 2018).

This leads to concerns over how permits are allocated. R19 pointed out that, *“The first step to China’s ETS is the design of initial permits allocation scheme and the solution to associated equity concerns.”* R16 aptly stated, “*It would be greatly difficult for China’s ETS to achieve equity and justice. As observed from the controversy existing in Intended Nationally Determined Contributions (INDC), it is crucial that various Chinese enterprises need to be persuaded for accepting their reduction targets and permits.”* R1 suggested that, *“Undeveloped Western areas should be compensated by the optimization of initial permit allocation and appropriate use of the revenues raised.”* R25 elaborated by saying, *“Most of the Western regions with lower marginal abatement costs have weak economic endurance, and also bear the responsibility for ecological environment protection. However, these situations are not typically included in the abatement costs nor reflected in the policies. Therefore, indirect costs within Western regions should be considered in the system design.”* R11 pointed out, *“As China’s ETS has decided to adopt the free distribution method, it must fully consider historical emission levels and the metric of GDP per capita so that more permits can be allocated to underdeveloped regions.”* However, R23 suggested that permit allocation should move closer toward auctioning, *“If the auction method is adopted, the underdeveloped areas will be more likely to be compensated by the revenue from an auction, thereby alleviating distortions.”*

Various allocation methods imply different emission reduction responsibilities or emission rights, potentially resulting in inequitable outcomes. Hence, *“There is a game of interests between the state-owned enterprises as well as between higher and lower levels of governments, because different allocation schemes might favour different interest groups,”* as R5 stated. Furthermore, due to a lack of historical emissions-related data, it is difficult to unify a baseline between existing enterprises and new enterprises (Qi and Wang 2013a). R7 affirmed this point by stating, *“Due to the lack of historical emission data, emission permits allocation schemes are defective, which leads to extremely low carbon prices that would seriously affects market activity.”*

Second, the implementation of China’s ETS will also require facilitating equitable trading in the carbon market (Zhang et al. 2014). A majority of experts attributed the inequitable nature of Chinese pilot programs to the imperfect laws and regulations pertaining to the systems’ operation. Because emission permits are public goods whose scarcity stems from mandatory emissions cap, the stable operation of an ETS cannot be separated from a complete legal and regulatory system (Zhou and Li 2019). R7 stated, *“Equity consideration should involve each link of the transaction procedure,”* also detailing that, *“A sound legal framework would not only clarify property rights, thereby avoiding market failures in the process of trading, but also provides a legal basis for punishment of unqualified enterprises so that it can raise the credibility of the system and guarantee the smooth operation of an ETS.”*

A majority of respondents were also concerned most about the lack of supervision, the relative immaturity of the Monitoring, Reporting, and Verification (MRV) system as well as the poor transparency of information. In this regard, R11 mentioned that, *“Although some ETS pilots have already accumulated experience of MRV, improving this capacity in support of a nationwide data management process will likely take more years with immediate effort.”* An effective and reliable MRV system is able to reflect the relationship between supply and demand in permit trading process and also effectively reflect the carbon price fluctuations (Zhao et al. 2016). Such signals are more likely to provide enterprises with a clear guidance for managing their investments. However, R8 stated, *“Presently, the ETS pilots in China is still imperfect, and there remain many deficiencies in the technical development of monitoring standards and facilities.”*

### The innovation dimensions of China’s ETS

Interestingly, the survey required participants to comment on the extent of innovation currently present within China’s ETS pilots and China’s future ETS, in terms of both trading institutions and trading procedures. Table 5 shows, perhaps surprisingly, that few respondents were able to identify *anything* innovative about the ETS, with one respondent marking that “*There is no innovation existing in Chinese pilot systems, it is still at the stage of imitation.”* Other respondents, however, discussed how they felt that China has implemented an innovative layered decomposition of binding indicators to form a relatively mature assessment mechanism, an issue also mentioned by (Lo 2013). In comparison to the market-based system, this assessment mechanism heavily relies on command-and-control regulations (Lo 2012). As such, as R1 put it, *“Organically combining the carbon trading market with the current assessment mechanism would be Chinese characteristics in the future.”*

**Table 5:** Identification of innovation aspects of China’s ETS mentioned by respondents (n=68)

Rank	Frequency by survey		Innovative institutions in China’s ETS
1	36	52.94%	No innovations/None
2	8	11.76%	From ‘Shidian’^1^ programs to the national carbon market
3	7	10.29%	At the stage of imitation
3	7	10.29%	Strong role of government
4	4	5.88%	Carbon financial derivatives
5	3	4.41%	Purpose of upgrading the industrial structure
6	2	2.94%	Chinese characteristics
6	2	2.94%	Unsure

Source: Authors.

^1^Shidian refers to pilot projects implemented prior to a national law being drafted, which is a well-recognized concept used in China’s policy making process

R6 suggested that the pilots themselves were innovative and intended to facilitate learning, noting that *“ETS pilots represent the spirit of Shidian.”* Shidian refers to pilot schemes that are normally operated in several selected areas prior to being promoted to the entire country. This method helps Chinese governments to identify challenges and avoid risks and thus assists the policy makers in eventually designing a unified program (Zhang et al. 2014). This process refers to the Chinese philosophy of *“Crossing the river by feeling the stones”* (Jotzo et al. 2018, P267). In this vein, R7 also remarked: *“On the basis of Shidian, a unified national ETS then is implemented.”* R14 quoted the Shenzhen pilot project as an example and commented, *“It draws lessons from the EU ETS’s allowance allocation and information system, the risk control of California’s trading market, the reduction of Tokyo’s urban construction emissions, and also the Australian carbon market’s strategy of fixed prices.”* In a similar way, R5 praised the same pilot scheme by stating, *“Shenzhen also demonstrates innovation in regard to trading regulations and trading processes, such as the introduction of a game mechanism and individual participants.”*

Other respondents discussed how an innovative feature of the Chinese ETS was its use not of an absolute reduction target, but instead of a rolling target coupled to carbon intensity as well as the country’s obligation under the Paris Accord. As R4 stated, *“China’s ETS has adopted the approach for reduction of carbon emission intensity between 2020 and 2030 instead of absolute targets”* An additional Chinese-specific feature mentioned as innovative was that electricity prices in China are administratively regulated, a point also examined by (Fan 2018). R33 commented on the issue by stating, *“Electricity price regulation tends to lead to a 20% to 30% increase in market price in comparison to unregulated conditions, which would, in turn, weaken the cost-effectiveness of the carbon market. Therefore, integration of the power industry into an ETS is an important challenge for the construction of China’s ETS.”* Gallagher et al. (2019) have argued that this market-based system allows for deeper electricity decarbonization while it is also able to solve the huge overcapacity in China’s power sector over the longer term. R33 supplemented, *“Yet, in the short-term, the national ETS is insufficient to induce major emissions reductions due to the narrow industrial coverage and the anticipated low carbon price.”*

A final theme of innovation was connected to the development of new innovative technologies that could be provoked or prompted by the national ETS. Many respondents remarked how the ETS could lead to disruptive low-carbon technology. R32 put this into the context of carbon capture and storage (CCS) by stating, *“The technology is able to collect CO*_*2*_ *and stores it underground. However, two major problems exist in relation to this technology. One is that CCS itself consumes a lot of energy, which also comes from fossil fuels. For example, a coal-fired plant can capture and store the CO*_*2*_ *it produced, but it will use an additional 30% of the fuel. Another controversy is that stored CO*_*2*_ *may be released into the atmosphere again.”* R32 also coupled the likely development of CCS with renewables: *“It is impossible to achieve the goal of the Paris Agreement without the use of CCS, and these two problems could be solved in the future. By then, CCS should be the primary method of emissions reduction if our eventual goal is to achieve near zero or even net negative emissions*. *Nevertheless, the main method for China within these decades regarding contributing to emissions reduction is still the development and utilization of renewables.”*

However, although 42.9% of respondents demonstrated positive attitudes toward the innovative abatement technologies, other respondents held either neutral or negative attitudes (41.1% and 16.1%, respectively). R3 noted that, *“It is generally acknowledged that technology will lead to a decline in carbon emissions, but the technological uncertainty would cause the less predictable carbon price in future trading process.”* In addition, R22 explained that, *“With the utilization of renewables, the contribution of China’s ETS to reducing emissions will be very small.”* R28 also estimated that, “*With the breakthrough of abatement technologies, the volume of emissions reduction for industries and regions will increase accordingly, assuming economic development is stable. If emission cap standards are not able to be adjusted aptly, it would jeopardize China’s ETS. Therefore, along with such uncertainties, the national ETS will inevitably need to be updated”.* By contrast, R14 positively pointed out that, *“Innovative abatement technologies will enable China’s ETS to enter a new state in which total volume for emissions reduction will decrease but the carbon price will increase.”*

### The public awareness dimensions of China’s ETS

Public awareness concerns center primarily on the broader social acceptance of a climate policy regime, program, or instrument. Here, previous work has  generally taken a more narrow view in examining social attitudes or stated preferences, with studies not looking at climate change or the ETS *per se* but very specific technologies, such as solar energy (Yuan et al. 2011), wind power (Yuan et al. 2015), shale gas (Tan et al. 2020), “new energy vehicles” (Du et al. 2018) and electric mobility (Sovacool et al. 2019), or nuclear power (Yuan et al. 2017), or sub-themes such as energy security (Bambawale and Sovacool 2011) or air pollution (Sun et al. 2016). Surveys looking at broader social acceptance for climate mitigation have found that being a member of the communist party slightly increases the acceptance of national mitigation measures, but none of the usual socio-economic characteristics (such as gender, age, income, or education) play a significant role in shaping preferences (Schwirplies 2018). Liu and Mol (2013) noted in their survey that rural residents were generally more supportive of renewable energy development given its positive impacts on the local environment. Such findings do not yield any direct insights into the public awareness dimensions of the ETS.

**Fig. 5:**
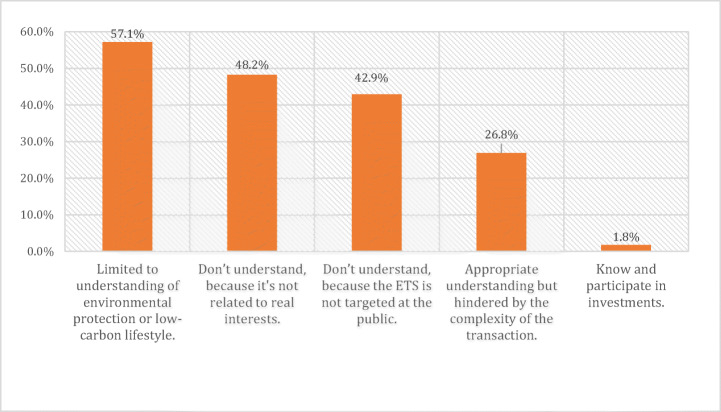
Survey responses reflecting on the public awareness barriers relating to China’s ETS (n=68). Source: Authors

Respondents mentioned repeatedly how few Chinese citizens recognize carbon emission trading or China’s ETS. As Fig. 5 reveals, an overwhelming majority of participants believed that the public is aware of environmental issues although few people recognize carbon emission trading. R9 reinforced this statement, *“Promoting an ETS in China lags behind that in Western states such as EU ETS and RGGI.”* Zhou and Li (2019) have indicated that Chinese respondents from government, enterprise and financial institutions lack awareness and knowledge of emissions trading. In addition, even more relevant entities such as industrial firms or energy technology suppliers have shown little enthusiasm for participation in the pilot systems and China’s ETS (Yang et al. 2016; Kang 2013). As a result, many Chinese enterprises remain unaware of the values of their carbon asset, which lead to excessive quotas and volatile prices (Liu et al. 2016; Zhou and Li 2019).

Additionally, R28 remarked that *“There are even some ETS pilots which simply enable specific enterprises or institutional investors to engage in trading.”* Zhang et al. (2016) warned that this could give rise to a limited number of trading participants and a low efficiency rating for the ETS pilots. R23 added, *“Although there was increased information released via pilot system, few people could accurately recognize carbon trading due to the complexity of theories and institutions.”* In addition, R22 mentioned that *“Chinese citizens’ awareness regarding emissions reduction is currently guided by government-led dissemination of information or campaigns.”*

That said, the results were not uniformly negative. R24 noted that, *“With the increased public awareness pertaining to environmental issues, the public increasingly tend to support environmentally friendly enterprises. Hence, enterprises will be more likely to participate in China’s ETS in order to gain social reputation.”* Klenert et al. (2018) have pointed out that recently ETS has been popularized with the primary goal of increasing public perception and acceptability under the Paris Agreement. However, due to the allocation method, the windfall profits in most ETS could be easily received by a small number of firms, which may be perceived as inequitable by the citizens (Klenert et al. 2018). Low carbon prices have also undermined public confidence in the scheme (Koch et al. 2016). To remedy this challenge, Zhao et al. (2016) and Qi and Wang (2013b) have suggested that ETS pilots should have set up a price floor and safety valve in support of decreasing uncertainties, but none of them has yet launched any specific measures.

A final aspect from our data concerns efforts to enhance public awareness of the ETS. Some respondents suggested that financial derivatives would attract additional individual investors (Table 6). Carbon financial derivatives differ from the traditional trading and include both the investment in green projects for reducing emissions and the financial intermediary activities with related financial regulations (Wang and Liu 2010). From this point of view, R12 noted that, *“The carbon market is the place for these types of transactions, which includes institutional and policy arrangements. Thus, establishing and developing an ETS is imperative for overcoming capital problem relating to low-carbon economic development.”* This idea, however, was contested. R11 warned that, *“If the public participates in carbon trading markets, the construction and operation of China’s ETS will face more challenges, such as the legality and validity of carbon assets, the financial supervision of carbon trading related activities and the training of ETS-related expertise.”*

**Table 6:** Ways for the public to participate in China’s ETS  identified by a subsample of respondents (*n*=15)

Rank	Frequency (by interview)		Feasibility schemes
1	10	66.66%	Carbon financial derivatives; individual investors
2	8	53.33%	Guided by enterprises; Ant Forest
3	6	40%	Voluntary emission reduction project
4	2	13.33%	Supervisors
5	1	6.66%	Mandatory participants

Source: Authors

Another prominent theme arising from our data relating to increasing attractiveness and awareness of the ETS is known as the Ant Forest game. This innovative digital product was developed by Ant Financial of the Alibaba Group to provide a game to enable users to be more environmentally conscious of their actions, with an initial emphasis on tree planting (Ant Financial Services Group 2018). R13 explained that, *“Ant Forest records and quantifies low-carbon behaviors in daily life, including walking, public transportation, and garbage classification. When emissions reduction volumes accumulate to 17.9 kg in the user’s carbon account, the user can plant a real tree via the Ant Forest platform.”* By the end of February 2019, users of the Ant Forest totaled over 350 million, which resulted in cumulative emission reductions exceeding 2.83 million tons with a total of 55.52 million trees being planted, accounting for an area exceeding 507 square kilometres (Global Times 2019). Thus, the Environomist (2017) has indicated that such innovative techniques have unique practical benefits in the global carbon market. As R31 puts it, *“There is a great chance that individuals will be included in the carbon trading market as a result of the guidance of internet companies via smartphones in the future.”*

## Future challenges on the horizon

Separate from the themes of equity, innovation, and awareness were future challenges that could affect the efficacy of the future ETS market. Respondents admitted that, currently, the newly devised national ETS is encountering considerable challenges and uncertainty regarding its future architecture. Table 7 demonstrates the top three significant challenges for China’s ETS from the survey, and the top ten reasons from the interviews. The most significant challenge facing China’s ETS is perhaps a lack of supportive government policies. Most experts expressed concerns about transitioning from regional pilot projects to the national unified system, primarily because of insufficient support policy, lack of relevant regulation and laws, the absence of unifying trade standards and of trained professionals in related fields. These concerns about the development of China’s ETS are associated with the expectations for its future architecture (Weng and Xu 2018).

**Table 7:** The challenges facing China’s future emissions trading scheme

Top panel: by survey (n=68)			
Rank question by survey			Challenge mentioned
1			Need for government’s support policies
2			Improvement of trading institutions and procedures
3			Transition from pilots to the national carbon market
Bottom panel: by interview (n=34)			
Rank	Frequency (by interview)		Challenge mentioned
1	25	73.52%	Transition from pilots to the national carbon market
1	25	73.52%	Insufficient support policy
2	22	64.70%	Imbalanced development of regions in China
3	20	58.82%	Linkage of pilots with the national carbon market
4	19	55.88%	Allocation schemes
5	15	44.12%	Absence of a legal system
6	12	35.29%	Unified national standards
6	12	35.29%	Impact of other reduction policies
7	9	26.47%	Need for top-level design
8	8	23.53%	Exogenous political, economic, and social factors
9	5	14.70%	Unclear position of this market-based policy
9	5	14.70%	Industrial coverage
10	2	5.88%	Lack of knowledge for both enterprises and the public

Source: Authors

Given the vastness of China’s territory and that different regions have varying economic development levels, resource endowments, and industrial structures, most experts have expressed their concerns about transitioning from regional systems to the national ETS. R1 added, *“The marginal abatement cost is higher in developed regions and lower in underdeveloped regions, such as the central and the Western regions. Such differences can be achieved even up to tens of times.”* R4 further stated that, *“It is difficult to achieve a unified and fair national standard due to imbalanced development. This can also be seen from the huge difference which exist in the current prices across the seven ETS pilots.”*

However, this striking disparity implies a significant difference in the marginal abatement costs across provinces (Cui et al. 2014). Therefore, China has significant potential to develop an ETS due to the fact that its marginal abatement cost is relatively lower than other emerging economies (Heindl and Voigt 2012). Similarly, R13 added, *“Despite the imbalanced development across the pilot projects, China’s ETS is still large enough to be the biggest of its kind, even if only the power sector is covered.”* In addition, R13 also noted, *“As a result of their diverse characteristics, each pilot market has conducted beneficial explorations into different dimensions of the national ETS.”* Thus, Lin and Liu et al. (2015) argued that the accumulated practical experience is particularly beneficial for exploring an effective mechanism, identifying problems, and optimizing system design, which can be considered as innovations for China’s ETS.

An additional and prominent concern that was mentioned was insufficient support policies. R27 was of the following opinion, *“The government’s determination is one of the major factors that would affect the development of the ETS. However, at present, this driving force alone is insufficient.”* In addition, R16 noted that, *“China is currently engaged in vigorous renewable energy development, which will remain the main driving force behind carbon emissions reduction for quite some time such that the ETS would make little contribution in the near future.”* To this, R12 stated that, *“By providing a national guidance, ETS infrastructure and institutions capacity would be developed more quickly.”* Indeed, Zhang et al. (2014) have demonstrated the fact that in the absence of a national law and unified standards, enterprises from various provinces have shown little enthusiasm and incentive to manage their activities. However, R2 added, “*The legal mandate for implementing an ETS on the national basis is far from clear. Many technical details of the new system’s design also remain unknown.”* Furthermore, several experts have realized that the promotion of these policies often lagged behind the plans. The official plan for China’s ETS shown in Table 1 that launched by NDRC in 2017 has proven the negative view. R30 warned about these risks by mentioning, *“Lagging policies typically weaken participants’ confidence in future markets and are not conducive for enterprises and investors who are planning their own carbon management strategy.”*

## Policy implications and recommendations

Based on an expert survey (n=68) as well as semi-structured expert research interviews (n=34), our data do give rise to a set of policy recommendations.

**Fig. 6:**
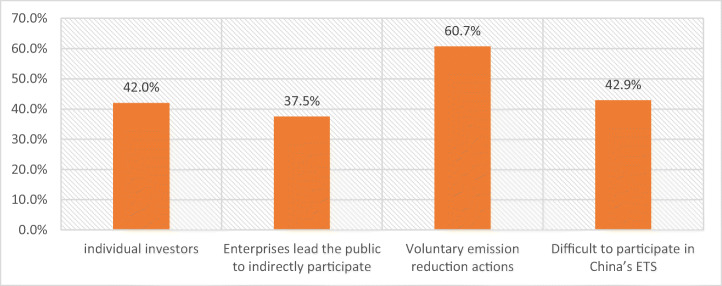
Ways for the public to participate in China’s ETS. Source: Authors.

First, adjustments to the allocation schemes and permit process within the ETS may be warranted. Our data strongly suggests that uneven economic and social growth could exacerbate any initial permits allocation scheme that would be a cornerstone for an ETS. In this regard, our respondents expressed a preference for allocation schemes that provide a lower emission-reduction burden on Western provinces and a relatively more significant one on the Eastern provinces. In addition, several experts also suggested that it is more equitable for China’s ETS to adopt an auction method instead of free distribution because as doing so will achieve an appropriate use of carbon revenues, which is likely to assist with alleviating distortions. Furthermore, a reliable and effective monitoring and verification system was stated as critical to create trust and confidence in the trading scheme. Figure 6 shows four specific suggestions from our data about how the public can better participate in the ETS, including investment, partnerships with enterprises, and voluntary actions.

Second, policy is needed to help hedge the substantial technological and institutional uncertainties that exist, which could potentially hamper the development of an ETS. Due to the development and utilization of renewables, some experts believe that the contribution of China’s ETS to emission reduction will be insignificant. Many other respondents questioned the strength and innovativeness of the institutions that would be involved in emissions trading. Although China’s ETS pilots have been ongoing for almost a decade now, respondents were still skeptical that sufficient learning had taken place. Stronger incentives for learning, as well as directed subsidies for low-carbon sources of energy including renewables but also efficiency, could promote this type of innovation, as could more government support for industrial-scale climate mitigation options, including carbon capture and storage or process optimization (Rissmann et al. 2020).

Third, low awareness (limited knowledge) or negative awareness (having oppositional attitudes or beliefs) among the public and private sector were identified as being significant barriers for ETS implementation. Although increased public awareness of climate issues does not necessarily lead to changes in individual behavior (DfT 2007), greater public acceptability of a climate policy is likely a crucial factor for environmental governance (Klenert et al. 2018). Aside from promoting awareness, most experts supported public involvement in China’s ETS via various schemes such as carbon financial derivatives and even an online game (known as Ant Forest).

## Conclusion

Because China leads the world in its total amount of carbon emissions, it is imperative that it designs an emissions trading scheme that is as equitable, innovative, and socially acceptable as possible. In order to meet the Copenhagen and Paris Accords, China has demonstrated a seemingly significant intention to establish a unified national ETS after 2020. The near-term future may very well be critical for China’s carbon emissions to peak, and also a formative but critical period for eventual low-carbon transformation.

At this stage, Chinese climate policy objectives are at a rare state of flux. The country has sought to implement measures to decarbonize its economy, constrain emissions, and meet its international pledges under the Paris Accord. At the same time, it is recovering from a global pandemic, managing growth in manufacturing and employment, and seeking to appease diverse stakeholder interests. Our results suggest that the road to an effective future ETS in China will likely be long and winding, with barriers that cut across many environmental, economic, and institutional dimensions. These must be proactively managed if the ETS is to get travelers to their ultimate and vital destination of emissions reduction—for China’s sake, and the world’s.

## Data Availability

The data used in this study is confidential.
